# Prevention and management of anastomotic leakage after colorectal resection: evolving trends among Italian surgeons. A SICCR survey

**DOI:** 10.1007/s13304-026-02625-7

**Published:** 2026-07-16

**Authors:** Renato Costi, Alfredo Annicchiarico, Antonio Amato, Cristina Folliero, Claudio Guerci, Alessandro Facchinetti, Filippo Montali, TabooSurvey Group, Adolfo Petrina, Agnese Dezi, Agostino Falcone, Agostino Fernicola, Alba Oliva, Alberto Buonanno, Alberto Porcu, Alberto Serventi, Alberto Stocco, Alberto Vannelli, Aldo Rocca, Alessandra Marano, Alessandro Baggi, Alessandro Bergna, Alessandro De Luca, Alessandro Soave, Alessia Fassari, Alessio Giordano, Alessio Morandi, Alex Bruno Bellocchia, Alfredo Annicchiarico, Alice Francescato, Amedeo Altamura, Amedeo Antonelli, Andrea Scardino, Andrea Balla, Andrea Barberis, Andrea Bondurri, Andrea Bottari, Andrea Divizia, Andrea Grego, Andrea Lauretta, Andrea Marco Tamburini, Andrea Martina Guida, Andrea Morini, Andrea Muratore, Andrea Picchetto, Andrea Pisani Ceretti, Andrea Rusconi, Andrea Tufo, Andrea-Pierre Luzzi, Angelo Alessandro Marra, Anna Canavese, Anna Guariniello, Annalisa Comandatore, Annalisa Pascariello, Antonella Nicotera, Antonella Usai, Antonella Veglia, Antonia Lavinia Zuliani, Antonino Agrusa, Antonio Amato, Antonio Azzinnaro, Antonio Bocchino, Antonio Cardarelli, Antonio Castaldi, Antonio Langone, Antonio Luberto, Arcangelo Picciariello, Armando Di Dato, Beatrice Pisillo, Beatrice Torre, Belinda De Simone, Biagio Picardi, Brunella M. Pirozzi, Bruno Nardo, Bruno Scotto, Bruno Sensi, Carla Tasca, Carlo Alberto Manzo, Carlo Giove, Carlo Salvemini, Carmen Sorrentino, Carolina Bartolini, Casoni Pattacini Gianmaria, Caterina Baldi, Caterina Foppa, Chiara Bettini, Chiara Casadei, Chiara Gatto, Chiara Marafante, Chiara Piceni, Christian Franzini, Claudio Cimmino, Claudio Guerci, Claudio Luciani, Colognesi Alberto, Cosimo Damiano Francione, Cristina Larotonda, Dajana Glavas, Daniela Daidone, Daniela Rega, Daniele Delogu, Daniele Fusario, Daniele Morezzi, Danilo Vinci, Dario Borreca, Dario Parini, Dario Somenzi, Daunia Verdi, David Alessio Merlini, Davide Cuneo, Davide Ferrari, Davide Mascali, Davide Montani, Davina Perini, De Flaviis Mattia, Dhimiter Cuka, Diego Coletta, Diletta Corallino, Edoardo Baldini, Edoardo Forcignanó, Edoardo Virgilio, Elena Martinuzzi, Elena Orsenigo, Eleonora Pozzi, Elisa Bolzoni, Elisa Galasso, Emanuele Damiano Luca Urso, Emanuele Pontecorvi, Emilio Paolo Emma, Enrico Pinotti, Erica Monati, Erika Boriani, Ernesto Tartaglia, Ester Marra, Ezio Lombardo, Fabio Ambrosini, Fabio Carbone, Fabio Marino, Fabrizio D’Acapito, Fabrizio Perrone, Fabrizio Romano, Federica Billeci, Federica Cavalera, Federica De Robertis, Federico Cozzani, Federico Festa, Federico Lovisetto, Federico Maggi, Federico Mariani, Federico Mazzotti, Felice Mucilli, Filippo Carannante, Filippo Montali, Francesca Abbatini, Francesca Albanesi, Francesca Ascari, Francesca Paola Tropeano, Francesca Pecchini, Francesco Belia, Francesco Bianco, Francesco Colli, Francesco Giovanni Cinieri, Francesco Matarazzo, Francesco Menegon Tasselli, Francesco Milardi, Francesco Pata, Francesco Persico, Francesco Salvetti, Francesco Tartamella, Gabriela Aracelly Arroyo Murillo, Gabriele Anania, Gabriele Ricci, Gabriella Lionetto, Gabriella Teresa Capolupo, Gaetano Luglio, Gennaro Giovine, Gennaro Mazzarella, Gerti Dajti, Giacomo Ambrogi, Giacomo Arcuri, Giacomo Carganico, Giacomo Franzini, Giacomo Fuschillo, Giacomo Mascheri, Giada Pattaro, Gian Andrea Binda, Gian Luca Baiocchi, Gianluca Baronio, Gianluca Cassese, Gianluca Fucci, Gianluca Garulli, Gianluca Mascianà, Gianluca Rizzo, Gianluca Vanni, Gianluigi Moretto, Gianmario Edoardo Poto, Gianpiero Gravante, Giorgia Gualtieri, Giorgio Lisi, Giorgio Rossi, Giovanna Pavone, Giovanni Alemanno, Giovanni Aprea, Giovanni Bellanova, Giovanni Castagna, Giovanni Cestaro, Giovanni Guglielmo Laracca, Giovanni Scudo, Giovanni Spiezio, Giovanni Tomasicchio, Giulia Citrigno, Giulia Germiniasi, Giulia Lauteri, Giulia Marini, Giulia Paradiso, Giulia Turri, Giuliano Barugola, Giuliano Lantone, Giulio Iacob, Giuseppe Curro, Giuseppe Frazzetta, Giuseppe Giuliani, Giuseppe Massimiliano De Luca, Giuseppe Navarra, Giuseppe Nigri, Giuseppe Palomba, Giuseppe Sica, Gloria Goi, Guenda Pulcina, Guglielmo Clarizia, Guglielmo Giannotti, Guido Sciaudone, Helen Yu, Iacopo Monaci, Ilaria Benzoni, Ilaria Clementi, Ilaria Ferrante, Imerio Angriman, Irene Fiume, Irene Tucceri Cimini, Jacopo Mercuri, Jacopo Weindelmayer, Juhye Jeong, Laura Fortuna, Laura Olivieri, Lavinia Piombetti, Leandro Siragusa, Leonardo Rossi, Leonardo Solaini, Letizia Malucchi, Letizia Santandrea, Linda Gabellini, Livio Iudici, Lorenza Beomonte Zobel, Lorenzo Arlia, Lorenzo Epis, Lorenzo Pagliai, Lorenzo Petagna, Lorenzo Vona, Luca Cardinali, Luca Cestino, Luca Domenico Bonomo, Luca Fabris, Luca Ferrario, Luca Mattia Quarti, Luca Morelli, Luca Perin, Luca Resca, Luca Salvador, Luca Scaravilli, Luca Sullo, Luca Tirloni, Luca Vicenzi, Ludovico Carbone, Luigi Cayre, Luigi Eduardo Conte, Luigi Fiorello, Luigi Marano, Luigi Percalli, Manuel Baldinu, Manuela Mastronardi, Marcello Pisano, Marco Anania, Marco Beggiato, Marco Clementi, Marco D’Ambrosio, Marco Giacometti, Marco Materazzo, Marco Pellicciaro, Marco Realis Luc, Marco Sparavigna, Margherita Carbonaro, Maria Carmela Giuffrida, Maria Francesca Chiappetta, Maria Roberta Fortunato, Maria Rosa D’Anna, Mariafelicia Valeriani, Marianna Capuano, Marianna Petrillo, Marina Valente, Mario Annecchiarico, Mario Giuffrida, Mario Pacilli, Mario Sorrentino, Marta Goglia, Marta Mozzon, Martina Bonafede, Marzia Franceschilli, Massimiliano Mistrangelo, Massimo Fedi, Matelda Bencini, Matteo Palmeri, Matteo Rossini, Maurizio Rho, Maurizio Romano, Maurizio Roveroni, Maurizio Zizzo, Mauro Filosa, Mauro Marzano, Mauro Montuori, Mauro Podda, Mauro Pozzo, Mauro Santarelli, Michela Campanelli, Michele Bellofiore, Michele Manara, Michele Manigrasso, Mirko Barone, Nicholas Rizzi, Nick Salimian, Nicola Cillara, Nicola Leone, Nicola Tartaglia, Nicole Josephine Castiglia, Nicolò De Santis, Nicolò Falco, Nicolò Pizzetti, Nicolò Turco, Nirvana Maroni, Oliva Giusepina, Omar Ghazouani, Oreste Claudio Buonomo, Pamela Milito, Paola Batistotti, Paola De Nardi, Paolina Saullo, Paolo Carcoforo, Paolo Massucco, Paolo Ossola, Paolo Pizzini, Paolo Vincenzi, Pasquale Losurdo, Pierluigi Angelini, Pierpaolo Sileri, Pietro Anoldo, Pietro Fransvea, Pietro Giorgio Calò, Raffaele De Filippi, Raffaele Galleano, Raffaele Lombardi, Renato Pietroletti, Riccardo Borreca, Riccardo Fratarcangeli, Riccardo Marsengo, Roberta Longhin, Roberta Tutino, Roberto Cammarata, Roberto Perinotti, Rocco Aversa, Rocco Pasqua, Rosita De Vincenti, Sabino Capuzzolo, Salvatore Buscemi, Salvatore De Maria, Samuele Vaccari, Sandro Giannessi, Sante Capitano, Sara Cecconi, Sara Crociato, Sara Gobbi, Sara Ingallinella, Sara Lauricella, Sara Vertaldi, Sebastian Tiso, Serena Molica, Sergio Sforza, Silvia Curcio, Silvia Negro, Silvia Neri, Simona Ascanelli, Simona Badalucco, Simona Pisicchio, Simone Buccianti, Simone Gargarella, Simone Guadagni, Simone Muratori, Sofia Esposito, Sophia Costacurta, Stefania Bettoni, Stefania Piccioni, Stefano Barbieri, Stefano Carini, Stefano Lafranceschina, Stefano Olmi, Stefano Rossi, Stefano Scabini, Teresa Perra, Tommaso Farolfi, Tommaso Fontana, Tommaso Iaquinta, Tommaso Loderer, Tommaso Violante, Ugo Grossi, Valentina Rampulla, Valentina Zucchini, Vania Silvestri, Vincenza Paola Dinuzzi, Vincenzo Adamo, Vincenzo Schiavone, Vincenzo Trapani, Vittoria Bellato, Vittoria Grammatico, Gian Andrea Binda

**Affiliations:** 1 Department of Medicine and Surgery, University of Parma, Parma, Italy; 2 Department of General Surgery, Vaio Hospital, Fidenza, Italy; 3 General Surgery Hospital of Imperia, Imperia, Italy; 4 Unit of General Surgery, Udine Hospital, Udine, Italy; 5 General Surgery Department, Luigi Sacco University Hospital, Milan, Italy; 6 General Surgery, Biomedical Institute, Genoa, Italy

**Keywords:** Anastomotic leak, Diagnosis, Management, Colorectal surgery

## Abstract

Anastomotic leakage (AL) remains one of the most feared complications after colorectal resection. Although several strategies have been introduced in the fields of AL prevention, diagnosis and management, their application in clinical practice is still to be assessed. A 26-item-questionnaire was administered between December 2023 and March 2024 to residents in general surgery and surgeons to analyse the actual clinical practice in Italy, in general and with respect of surgical experience in colorectal surgery. A total of 414 responses were analyzed. Most respondents were general surgeons/residents working in public or university hospitals; over one-half had limited surgical experience (< 50 colectomies). Regarding low colo-rectal anastomosis techniques, Knight-Griffen technique (44.0%) and hand-sewn colo-anal anastomosis (37.4%) were the most commonly reported. “Preventive” measures included reinforcement sutures (29.7%) and dog-ear avoidance procedures (21.7%); among intraoperative tests, hydropneumatic leak test (94.9%), “rings’” check (87.9%) and indocyanine green fluorescence (61.8%) were widely used. Protective stomas were created selectively in 52.4% of cases, with ileostomy largely preferred (93.9%). Despite ERAS recommendations, drains were frequently placed, particularly after anterior resection (73.7%). In the case of paucisymptomatic leak, conservative management was preferred (73.4% for ileo-colic, 65.9% for colorectal), at times associated with endoscopic procedures (15.2%). Decisions regarding diverting stomas or Hartmann’s proedures were mainly driven by clinical presentation rather than AL size. Endoscopic treatments, especially intracavitary “sponge” placement, have gained widespread acceptance. This survey highlights partial adoption of current guidelines. Although traditional practices, including drainage placement, persist, conservative and endoscopic approaches are increasingly adopted in the management of AL.

**Supplementary Information:**

The online version contains supplementary material available at 10.1007/s13304-026-02625-7.

## Introduction

Anastomotic leakage (AL) is a specific, feared early complication of colorectal resection and is responsible of significant morbidity and mortality, ranging 20–35% [1, 2], and 2–16% [2–5] respectively.

AL occurrence and its severity have been associated with several factors, including operative setting (election/emergency), surgical indication, type of resection and site of the anastomosis (right colectomy, transverse colectomy, sigmoidectomy-left colectomy, anterior resection) [6–10], comorbidities and systemic therapy, including immunosuppression/diabetes, and voluptuous behaviours, such as cigarette smoking [3, 9, 11, 12].

With the purpose of reducing AL rate, procedures and maneuvers aimed at verifying the appropriateness of the anastomosis’ technique and the correct vascularization of anastomotic stumps have been introduced, including the administration of indocyanine green [13, 14].

Anastomosis technique has also evolved through years, with an increasing role played by mechanical stapling [15–17], and still nowadays a preeminent role is played by double stapling procedure, so-called Knight-Griffen technique [18]. Several techniques, manoeuvres and tricks have been proposed to reduce AL rate [19–22]. 

Moreover, the use of perianastomotic drains achieve an AL early diagnosis and to potentially temper its consequences, is nowadays challenged by recently introduced fast track protocols, including ERAS [23], but is seemingly still largely adopted.

In the case AL eventually occurs, its management is also evolving, with several options alternative to traditional re-do or salvage surgery such as mini-invasive laparoscopic lavage/drainage, imaging-guided radiologic techniques [24–29] and endoscopic procedures, including trans-anastomotic drain positioning, stenting, and suturing/clipping [30–32]. Last, colonic transit diversion by (ileal or colonic) stoma has still a pivotal role in left-sided colorectal resections, as it is traditionally seen as mean of AL “prevention” if performed intraoperatively (mostly in anterior resections) or as a possible treatment of significant but not major postoperative AL [9, 29, 33, 34].

Although all those efforts, AL did not find any definitive solution over the past five decades and remains the main issue of colorectal resective surgery [35, 36], as AL is still reported in 3–8% of cases overall [37, 38], and with even higher rate after low rectal resections [9, 39]. 

In the last two decades, guidelines [40–42] and scoring systems [9, 43–45] have been proposed in order to define the correct preparation, technique and management of colorectal surgery, and to identify patients at high risk of developing an AL, to be possibly managed differently. Unfortunately, surveys based on national registry databases [46–49] show that clinical practice is not evolving as generally recommended, probably also owing to several factors, including difficult AL multidisciplinary management in an emergency setting, unavailability of latest technology in peripheral hospitals, and an increasing trend towards defensive medicine by general surgeons during their duties.

The present survey is aimed at an appraisal of actual clinical practice in AL prevention, diagnosis and management in the early 2020s, in order to verify the real spread of recent recommendations and the progressive abandonment of nowadays unjustified behaviors. As a secondary purpose, the present analysis is aimed at identifying different attitudes associated with clinical experience, by stratifying survey responders in young (residents/within 5 years from residency) and expert surgeons.

## Methods

The survey was carried out between December 2023 and March 2024 by the Colorectal Emergency Section of the Italian Society of Colorectal Surgery (SICCR) and the data were collected using an online questionnaire by Google Forms. This survey, named “Taboos in emergency colorectal surgery – Section: anastomotic leak”, aims to explore surgeons’ attitude in AL prevention, diagnosis and management, either in an elective or emergency setting, in real life, regardless of the currently available guidelines and recommendations. The purpose of the survey was explained to all respondents with a brief introduction, and respondents were asked to sign a privacy policy consent on a voluntary basis. Both general surgery residents and certified surgeons with various experience in general and colorectal surgery were considered eligible for the survey.

**Fig. 1:**
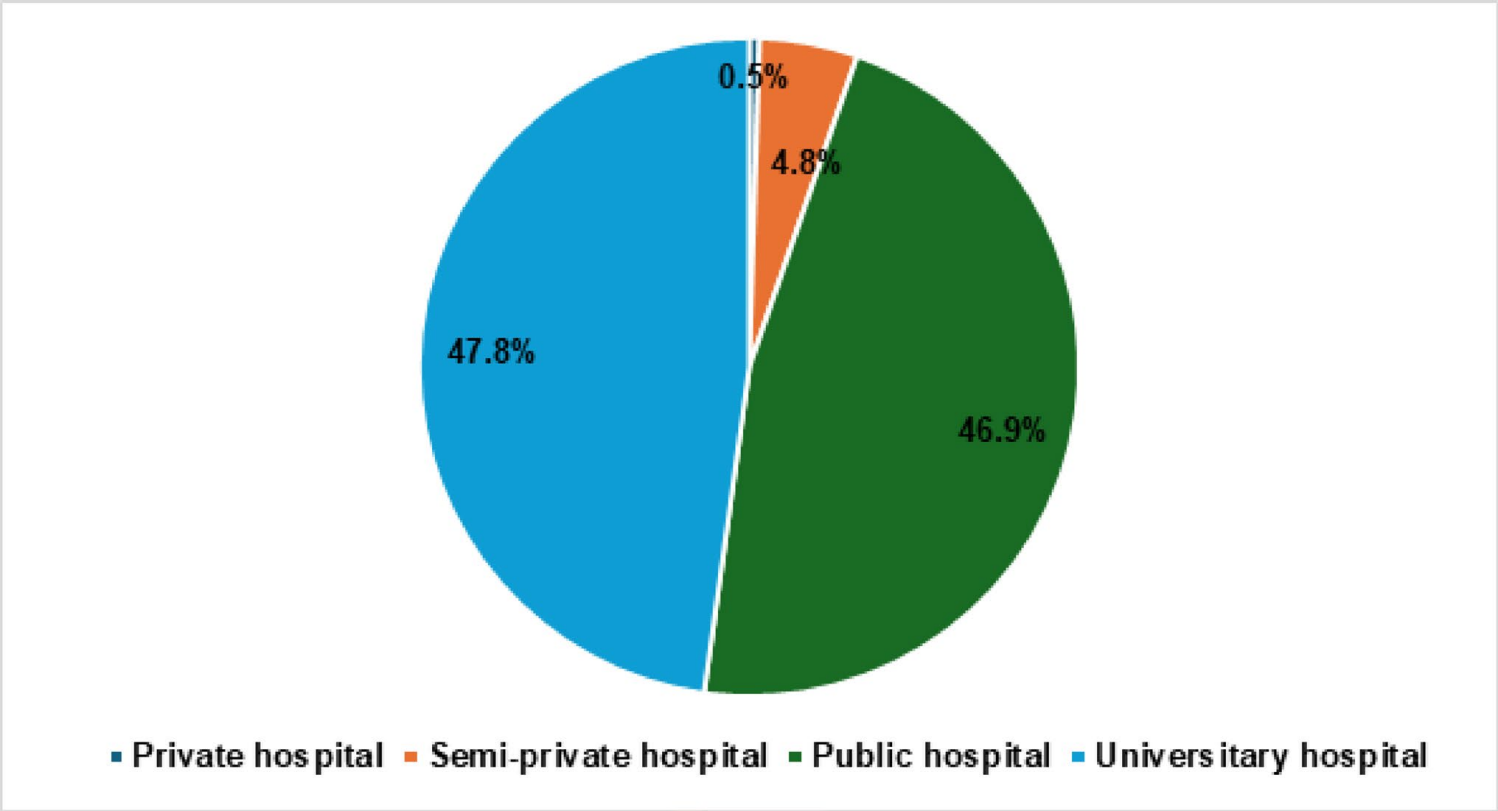
Type of hospital. Distribution of the various hospital settings among the respondents.

Responders were invited to answer a 26-item-questionnaire divided into six sections: General Information, workplace and personal experience in colorectal surgery (Q1-Q10); Technique of low anastomosis (Q11); Intraoperative “tricks & checks” for leak prevention (Q12-Q13); Indication and technique for stoma (Q14-Q16); Drain management (Q17-Q19); Leak management (Q20-Q26) (Table 1).

Answering to all questions was mandatory to complete the survey. Survey administration to surgeons was performed by mailing lists, instant message services, and the official social media accounts of the Italian Society of Colorectal Surgery (Società Italiana di Chirurgia ColoRettale, SICCR) on Facebook, Instagram, and LinkedIn. A reminder was mailed two, four and six weeks after the first mailing. All respondents were informed that the results of the survey would have been used for further statistical evaluation and scientific publication. Anonymity was guaranteed by study design. After the closing date for questionnaire submissions, results were downloaded as a comma separated values (CSV) and analysed by using Excel (Microsoft Corporation, Redmond, USA). Results of the survey were reported according to the Checklist for Reporting Results of Internet ESurveys (CHERRIES) guidelines [50].

### Statistical analysis

Collected data were processed, and results were summarized as frequencies (no.) and percentages (%), separately for each question. A further stratification of the outcomes was obtained dividing the responses into one class of experience according to number of colectomies performed (≤ 50 vs. ≥ 51). All the data were reported in contingency tables for subsequent inferential analysis. Statistical analyses were performed using the commercial software “SPSS” (IBM SPSS Statistics for Windows [Version 28]. Armonk, NY: IBM Corp.), the open source statistical system “R” (R Core Team. (2023). R: A language and environment for statistical computing. R Foundation for Statistical Computing, Vienna, Austria. URL: https://www.R-project.org/), and the freeware package of statistical programs for epidemiologists “Winpepi” (Abramson, J. H. (2016). WinPepi: Computer programs for epidemiologists. [Version 11.65]. (Retrieved from http://www.brixtonhealth.com/pepi4windows.html).

The contingency tables obtained from each question were analyzed using the chi-squared test and the Fisher’s exact test, as appropriate, in order to assess the potential difference between experience groups. The results were considered statistically significant for a p-value below 5% (*p* < 0.05).

## Results

### General information, workplace and experience in colorectal surgery (Tables 2, 3 and 4; Figs. 1, 2 and 3)

**Fig. 2:**
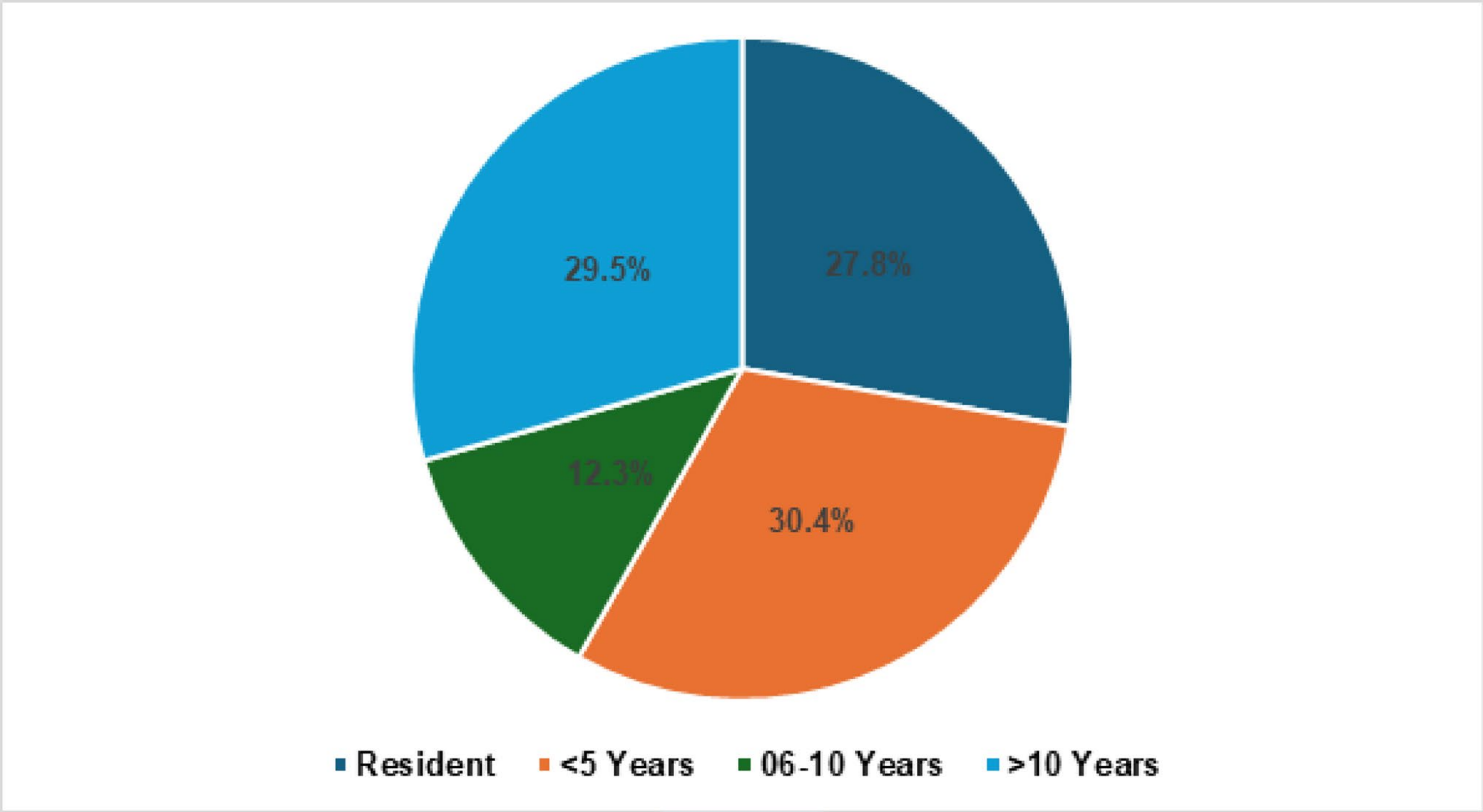
Surgeon personal experience (years). Years of experience among the respondents.

**Fig. 3:**
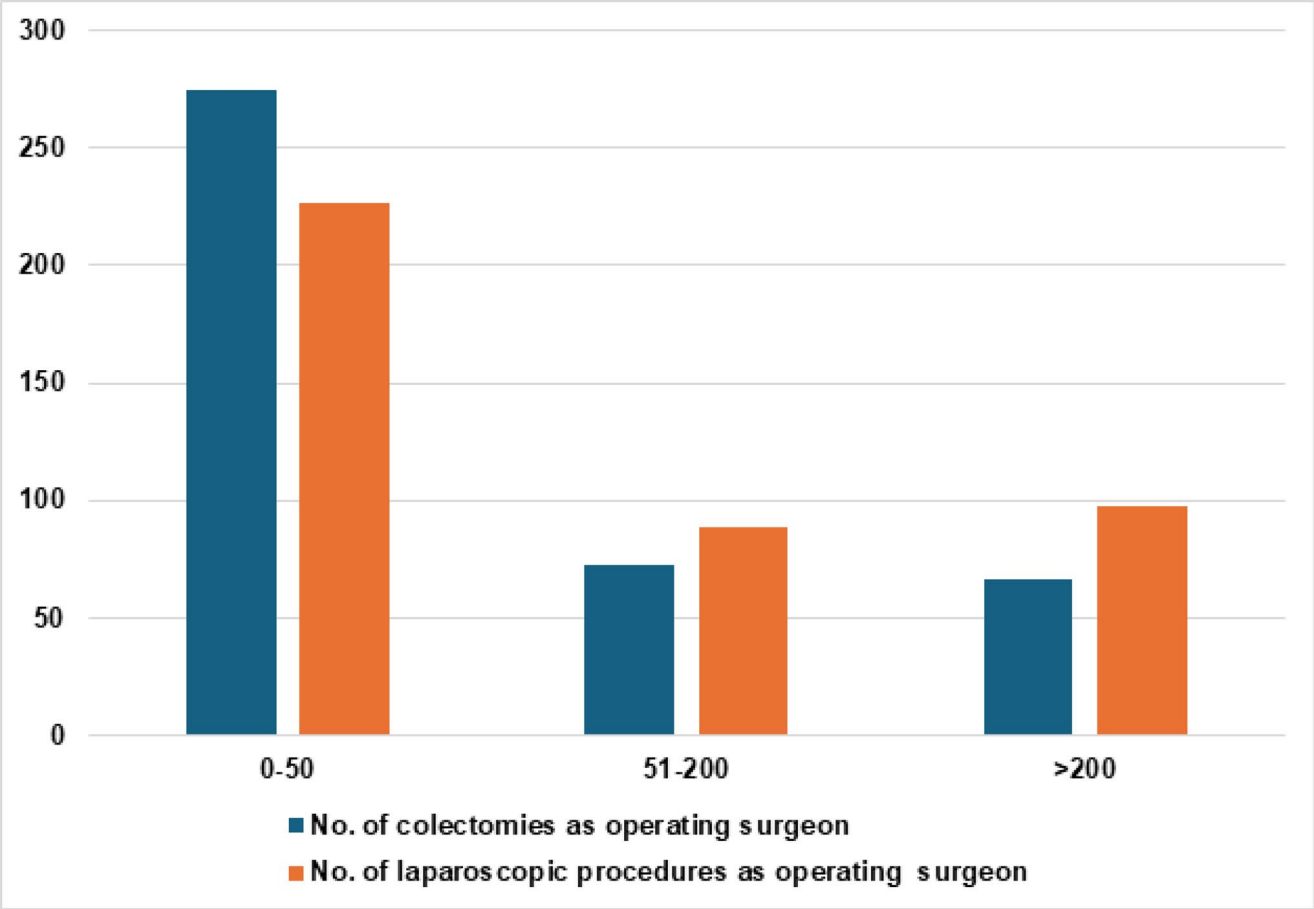
Surgeon’s operative experience (colorectal, laparoscopy). Number of colectomies and laparoscopic procedures performed by respondents.

Five hundred and ten questionnaires were correctly completed. After excluding 96 double reports from the same responders, questionaries from 414 different respondents were considered eligible for the final analysis. Overall, 286 (69.1%) respondents were men and 128 (30.9%) women. Almost all the interviewees worked in public hospitals (one half of them in University hospitals (198; 47.8%) (Fig. 1). In most environments, an intensive care unit (400; 96.6%) and a regular activity of emergency surgery (377; 91.1%) and interventional radiology (321; 77.5%) were present (Table 2). Table 3 highlights the Italian working regions of the respondents to the survey (Table 3). Concerning surgical experience, more than half of interviewed surgeons (115; 27.8%) were in training program or within 5 years from the end of residency, whereas 122 (29.5%) had more than 10 years of surgical experience (Table 4-Figure 2). 66.4% (275) had performed fewer than 50 colonic resections and 23.7% (98) had performed more than 200 laparoscopic procedures (Table 4; Fig. 3).

## Technique of low anastomosis (Question 11; Table 5)

Overall, the most frequently performed procedure was the Knight-Griffen technique (44.0%), followed by manual colo-anal anastomosis (37.4%). Less common approaches included pull-through procedures (8.7%), Transanal Transection and Single-Stapled Anastomosis (TTSS) (8.0%), and other techniques (1.9%). When stratified by surgical experience, no significative differences were observed. Among less experienced surgeons (0–50 colectomies), the Knight-Griffen technique was reported in 47.3% of cases, while manual colo-anal anastomosis accounted for 36.0%. In the expert group (> 50 colectomies), manual colo-anal anastomosis was slightly more frequent (40.3%) than Knight-Griffen (37.4%). The rate of pull-through and TTSS procedures, rarely used overall (10.8% vs. 7.6%, and 9.3% vs. 7.3%, respectively) was similar in the two groups. The comparison between groups did not show statistically significant differences (*p* = 0.391) (Table 5).

## Intraoperative “Tricks & Checks” for leak prevention (Questions 12–13; Table 6)

Regarding leak prevention, the most common approach was to avoid any specific measures (38.9%), followed by anastomosis’ reinforcement sutures (29.7%) and techniques to prevent dog-ears (21.7%). Less frequently, surgeons reported the use of glue (5.6%) or other methods (4.1%). Considering surgical experience, although difference between groups did not reach statistical significance (*p* = 0.098), surgeons with > 50 colectomies were more likely to avoid any leak prevention measure (45.3% vs. 35.6%), whereas reinforcement sutures (32.0% vs. 25.2%) and dog-ear prevention (23.6% vs. 18.0%) were more frequent among less experienced surgeons. The use of glue remained uncommon in both groups, while other strategies were slightly more frequent among highly experienced surgeons.

Concerning intraoperative leak checks, the hydropneumatic leak test was the most widely used method (94.9%), followed by the rings’ check (87.9%) and indocyanine green (ICG) fluorescence (61.8%). Only a minority of surgeons reported performing no test (0.7%) or using other techniques (4.3%). No relevant differences were observed between groups in terms of the hydropneumatic leak test or omission of testing. Rings’ check and ICG use showed roughly the same rate among more experienced surgeons (89.2% vs. 87.3% and 67.6% vs. 58.9%, respectively). No statistically significant differences were observed between the two groups overall (*p* = 0.866) (Table 6).

### Indication and technique for stoma (Questions 14–16; Table 7)

In patients undergoing anterior resection with double-stapling mechanical, colorectal anastomosis (Knight–Griffen technique), the majority of surgeons reported a non-systematic approach, performing a protective stoma only when indicated by local or patient-related conditions (52.4%). Systematic protection was reported in 25.9% of cases when the anastomosis was < 4 cm from the anal verge, in 15.2% when < 8 cm, and in 6.0% when < 12 cm. Only 0.5% of respondents reported to never perform a protective stoma. When stratified by surgical experience, less experienced surgeons more frequently opted for a non-systematic approach (57.1% vs. 43.2%), whereas more experienced surgeons showed a preference for systematic protection in the case of anastomosis performed at < 8 cm (21.6% vs. 12.0%). The difference between groups reached statistical significance (*p* = 0.023) (Question 14; Table 7).

Regarding ostomy technique, ileostomy 15 cm from the ileocecal valve (ICV) was the most performed (53.1%), followed by ileostomy 30 cm from the ICV (40.8%). Other techniques such as left colostomy (2.7%), right transversostomy (2.2%), and other approaches (1.2%) were rarely adopted. No significant difference was observed between surgeons with different levels of experience (*p* = 0.212) (Question 15; Table 7).

Other than the anastomosis’ distance from the anal verge, several clinical and technical variables influenced the decision-making process. The most frequently reported factors were local conditions such as tissue inflammation (84.5%), preoperative radiotherapy (84.1%), and the quality of the anastomosis (70.3%). Additional criteria included other comorbidities (61.8%), BMI (49.3%), type II diabetes mellitus (40.6%), and smoking habits (30.4%). Although no differences were observed between groups, type II diabetes mellitus, when evaluated alone, resulted as being more considered by less experienced surgeons (57.5% vs. 7.2%), with a statistically significant difference (*p* < 0.001) (Question 16; Table 7).

## Drain management (Questions 17–19; Table 8)

The survey investigated surgeons’ practice regarding drain placement after various operations including colectomies and anterior rectal resection.

After right hemicolectomy, most surgeons reported not to place a drain (36.7%), followed by placing a retroanastomotic drainage (18.4%) and in the right parietocolic gutter (17.4%). A further 16.2% reported avoiding drainage only in patients following the ERAS protocol, whereas 7.0% placed one drain in the pelvis and 2.9% systematically placed two drains. No statistically significant differences were observed between less and more experienced surgeons (*p* = 0.401) (Question 17; Table 8).

After left hemicolectomy, most surgeons opted for a drain in the pelvis (49.0%), followed by no drain at all (17.9%) and drain placement in the left parietocolic gutter (12.8%). Systematic use of two drains (5.1%) and avoidance of drainage in ERAS patients (8.9%) were less common, while only 5.3% of respondents limited drain use to patients with comorbidities. Surgeons with > 50 colectomies were more likely to avoid drains (26.6% vs. 13.5%), with a statistically significant difference between groups (*p* = 0.044) (Question 18; Table 8).

After anterior resection of the rectum, the vast majority reported placing a drain in the pelvic cavity (73.7%), followed by the systematic placement of two drains (11.8%). “No drain” was reported by 5.8% of respondents systematically, while 3.4% omitted it only in ERAS patients, and 3.1% limited drain use, to patients with comorbidities. Other strategies and alternative locations were rarely mentioned. No significant differences were found between surgeons with different experience (*p* = 0.714) (Question 19; Table 8).

## Leak management (Questions 20–26; Table 9)

The management of anastomotic leaks varied according to clinical presentation and surgeon experience. In the case of ileo-colic anastomosis with a drained clinically paucisymptomatic AL, most surgeons opted for a conservative approach and CT imaging (73.4%), while 15.9% performed laparoscopy, 6.3% chose endoscopic exploration, and only 3.4% preferred laparotomy. Surgeons with ≤50 colectomies were more likely to choose a conservative approach (76.7% vs. 66.9%), whereas laparoscopy (20.1% vs. 13.8%) and laparotomy (6.5% vs. 1.8%) were more frequent among experienced surgeons, with a statistically significant difference (*p* = 0.023) (Question 20; Table 9).

Considering the reoperation for ileo-colic AL up to 2 cm with localized peritonitis, the preferred options were ileocolic re-anastomosis without protective stoma (36.0%) and ileocolic re-anastomosis with stoma (29.5%). Raphia with ileostomy was reported by 23.7%, while terminal stoma creation (7.2%) and simple raphia (2.4%) were less frequent. Although less experienced surgeons more frequently chose re-anastomosis with stoma (32.4% vs. 23.7%) and more experienced surgeons preferred re-anastomosis without diversion (44.6% vs. 31.6%), such differences did not reach statistical significance (*p* = 0.104) (Question 21; Table 9).

Concerning patients with colorectal anastomosis with a clinically paucisymptomatic, drained AL, conservative treatment was the most common choice (65.9%), followed by endoscopic exploration (15.2%) and laparoscopy ± stoma (13.3%), with very few cases managed by open stoma creation (2.7%) or laparotomy (0.5%). No significant differences were observed between groups (*p* = 0.894). (Question 22; Table 9).

When asked when to perform a diverting ileo/colostomy in the presence of a drained colorectal AL, most surgeons reported that the decision depended on clinical findings (52.4%). Other criteria included the extent of the leak, with thresholds > 1/3 (15.2%), > 1/4 (9.2%), or > 1/2 of the circumference (8.2%), while 13.5% opted for diversion systematically. More surgeons with > 50 colectomies reported to rely on clinical finding (61.1% vs. 48.0%), but the difference was not statistically significant (*p* = 0.056) (Question 23; Table 9).

In the case of a leak on postoperative day 6 after low anterior resection with Knight-Griffen anastomosis, the most frequent salvage procedure was ileostomy 15 cm from the ileocecal valve by laparoscopic approach (41.3%), followed by ileostomy 30 cm from the ileocecal valve with laparoscopy (32.4%). Other approaches included right transversostomy (11.9%), ileostomy with open approach (11.6%), and miscellaneous techniques (2.9%). No significant differences were found between groups (*p* = 0.133) (Question 24; Table 9).

When asked in which situation a salvage Hartmann’s procedure in the setting of a drained colorectal leak, 63.1% of surgeons reported taking their decision based on clinical presentation, whereas 30.2% of surgeons decided considering leak size: 19.6% for leaks > ½ of the circumference and 10.6% for leaks > 1/3 of the circumference. Only 1.9% performed Hartmann’s systematically. The overall comparison did not show any significant difference (*p* = 0.365) (Question 25; Table 9).

Finally, considering the management of drained AL involving up to 1/3 of anastomosis’ circumference, endoscopic management was preferred by the majority. Endoscopy options included intracavitary sponge placement (56%), associated with (30.9%) or without stoma (25.1%), followed by clips placement (17.4%) and endoscopic suturing (7.3%), whereas 19.3% did not consider any endoscopic approach. No statistically significant differences were observed between groups (*p* = 0.820) (Question 26; Table 9).

## Discussion

### Participants and participants’ facilities (Tables 2, 3 and 4; Figs. 1, 2 and 3)

Four-hundred-ten general surgeons/general surgery residents were included in the survey, with a > 50% rate of young surgeons, including 27.8% residents and 30.4% within 5 years from residency program. Experience in both major colorectal resections and laparoscopic surgery reported by survey responders are consistent with the young age of most participants. The geographical distribution of participants is related to the numerosity of population in each Italian region, with slightly higher rates of responders in Lombardia (17.9%), Emilia-Romagna (15.9%), Lazio (12.1%) and Toscana (10.9%), probably also owing to survey promotion activity by the authors (Table 3).

Many responders’ hospitals are secondary-tertiary general hospitals, as only roughly 5.3% were private health facilities (22/414), and only 8.9% without emergency surgery activity (37/414). Interestingly enough, almost ¼ of responders’ hospitals (93/414) does not have any interventional radiology unit available on site, which may be supposed to affect AL management, in particular in the case of limited peri-anastomotic abscesses potentially suitable of imaging-guided drainage. The number of University hospitals seems over-represented as they account for almost 50% (198/414), whereas the majority of surgical facilities in Italy are non-University institutions. Such a finding seems related to the high rate of residents (115/414; 27.8%), mostly attending University hospitals, completing the survey (Table 2; Fig. 1).

Consistently with those figures, the majority of responders have limited experience in colorectal surgery (275; 66.4% - has < 50 colectomy performed as operating surgeon) and laparoscopy (227; 54.8% - has < 50 performed laparoscopic procedures) (Table 4; Figs. 2 and 3). It is surprising that more than 2/3 of responders are men, since in Italy a recent trend towards a 1:1 gender ratio is ongoing. Since this trend is recent, this may explain such figures as well as the apparent significantly lesser experience in colorectal surgery reported by women. Age also influences colorectal experience, as the majority of surgeons > 10 years from residency program report to have performed > 50 colectomies, falling to less than 40% in the 6-10-y-span and less than 15% within 5 years from residency program. Those data should lead to some reflections in surgeons’ education through their early career, as > 90% of young surgeons with on call duties in hospital with emergency activities responding to the survey seemingly has limited experience in colorectal surgery. Interestingly, no difference emerged in surgeons’ colorectal experience by type of hospital (public/private) as well as by presence of third level services (ER, ICU, interventional radiology), showing the spread of colorectal surgery among Italian general surgeons, also outsides specialized centres.

### Surgical anastomosis and leak prevention techniques (Questions 11–13; Tables 5 and 6)

#### Technique for very-low colorectal anastomosis (≤ 2 cm from the dentate line)

As expected, surgeons’ body is conservative regarding anastomosis’ technique: the vast majority of them favours manual or mechanical (Knight-Griffen) colo-anal anastomosis, with less than 10% switching to the recently introduced TTSS [21] or any pull-through technique [51–54], which has been recently re-proposed (preferred by 33 and 36 responders, respectively). Colorectal experience stratified analysis did not show significant differences.

### Anastomosis’ technical “tricks”

The majority of surgeons add some “tricks” to his approach, mostly a “reinforce” suture (123; 29.7%) and some technical variants in order to avoid the so-called Knight-Griffen’s “dog-ears” (90; 21.7%), the lateral parts of double-stapling suture, which are diffusely perceived as the Achille’s heel of that technique due to supposed inadequate vascular supply. There is a trend towards not doing anything after colorectal anastomosis by more expert surgeons, whereas younger colleagues more frequently report technical variants/tricks, maybe because they mostly attend tertiary/University hospitals.

### Intraoperative anastomosis’ “checks”

Among the manoeuvres performed in order to check the correctness of surgical technique and stumps’ blood supply, the idro-pneumatic test and the so-called “donuts” check are almost universally performed, whereas ICG test is rapidly spreading and entering surgeons’ routine, as at the present time it is already performed by almost 2/3 of surgeons. Colorectal experience associated with stratified analysis did not show significant differences.

### Indication and technique for stoma (Questions 14–16; Table 7)

#### Stoma after anterior resection

Although protective stoma is traditionally performed by Italian surgeons after rectum anterior resection, the result of the survey is somehow surprising, as seemingly only 25 (6%) of surgeons perform a stoma systematically after anterior resection (regardless the distance from the anal verge of anastomosis), whereas more than 1/2 decides based on local findings and general conditions of the patient. To be fair, such a question may have been misleading as, overall, another 170 surgeons reportedly perform a stoma routinely after low anastomosis (< 4 or < 8 cm), and the answer “not systematically” actually could imply the distance from the dentate line as one of considered criteria.

From the comparison between less and more experienced colorectal surgeons, we may deduce that younger colleagues are more oriented towards a tailored use of protective stoma, as they are more likely to perform stoma “not systematically”, which is coherent with recent literature on the subject [55] whereas experience may induce a more prudent behaviour.

### Stoma techniques

Consistently with general recommendations [20, 56–58], ileostomy (389; ∼94.0%) is widely preferred to colostomy in order to temporarily protect high-risk colorectal mechanical anastomoses; in half of cases performed 30 cm from the ileocecal valve in order to allow a comfortable closure at a distance from the colorectal resection.

### Criteria for stoma

Local conditions such as tissue inflammation and previous radio-therapy are considered by over 80% of surgeons; suboptimal surgical technique (multiple “firing” by linear stapler) and general comorbidities or obesity are evaluated by 50–75%, whereas DM and cigarette smoking are not considered as factors by the majority of surgeons. This latter data is not consistent with recent literature and recommendations on the subject [8, 9]. Interestingly, DM is significantly more often ignored by more experienced surgeons in deciding whether to perform a stoma or not. Seemingly, younger colleagues are more up to date with international recommendations [9]. 

### Drain management (Questions 17–19; Table 8)

#### Overall

Also owing to the spread of fast-track programs (including ERAS), the use of intraabdominal drains after colectomy is progressively reducing especially for right-sided procedures. In fact, considering drain placement with respect of resected segment, a clear anticlockwise trend towards avoiding any perianastomotic drain from rectum anterior resection to left side resections to right colectomy, where it has been abandoned by roughly one half of surgeons.

#### Right colectomy

After right colectomy, perianastomotic drains are less and less placed by surgeons, roughly reaching 50%, owing to several reasons. First of all, a perianastomotic drain after right colectomy is de facto placed in the middle of peritoneal cavity, where it may easily move for the original perianastomotic position, also owing to the recovery of small bowel peristalsis; [59, 60] second, the liquid state of stools passing through an ileo-colonic leak and diffusing in the middle of the abdomen questions the usefulness of a drain itself as a “therapeutic mean”, as immediate emergency endoscopy/surgery seems the most appropriate management; [61] third, ERAS recommendations suggest to avoid drain placement to enhance recovery after surgery [23]. 

### Left colectomy

After left colectomy, at least one drain is still placed systematically by about ¾ of surgeons against ERAS recommendations. Such a drain is seemingly still nowadays diffusely considered helpful for leak early diagnosis, as well as a therapeutic mean for not severe leakage, possibly managed conservatively (endoscopic procedures, including stenting, sponging, suturing, clipping,…). Interestingly enough, more expert surgeons do not systematically place any drain after left colectomy, as they probably adapt their attitude on a case-by-case basis, in accordance with their experience.

#### Anterior resection

After anterior resection, the attitude of over 90% Italian surgeons remains to place at least one drain, usually in the Douglas (or perianastomotic) pouch. Significantly, in the case of anterior resection, ERAS recommendations suggesting avoiding abdominal drains are mostly ignored, as they are followed by just 14 surgeons (3.4%), whereas, on the contrary, as many as 2 drains are left in place by 49 surgeons (11.8%). The actual risk of leak after colorectal anastomosis, as well as the potential role of drain in the conservative treatment of non-severe leakage together with endoscopic management and antibiotic therapy, probably play a role in such an ingrained, die-hard habit.

#### Leak management (Questions 20–26; Table 9)

##### Clinically pauci-symptomatic leak of an ileo-colic anastomosis

Significantly, a conservative attitude is adopted by roughly ¾ of surgeons, in few cases associated with colonoscopy (and potentially endoscopic manoeuvre), whereas in just one out of 5 cases (80/414; 19.3%), surgery (by laparotomy or laparoscopy) is preferred. Significantly, higher percentage on non-conservative management is recorded among older surgeons (*p* = 0.023), when experience may suggest a more pondered management.

### Emergency surgery for leak of an ileo-colic anastomosis

Interestingly, when surgery is eventually needed for an ileo-colic leakage, 2/3 of surgeons (271; 65.5%) prefer a re-do anastomosis, associated or not with a stoma, rather than a suture of the leak (w/without stoma). Deteriorated tissue conditions due to localized peritonitis caused by ileal leak seemingly plays a role in such an attitude.

### Management of a clinically pauci-symptomatic leak of a colo-rectal anastomosis

Significantly, overall, over 80% of surgeons prefer a non-operative management, in just 15.2% (63/414) of cases associated with endoscopic assistance. In the remaining cases a stoma is preferred, mostly after laparoscopic exploration. The confidence in drain placement as an effective management of minor leaks in non-significantly symptomatic patients may be the reason of such persistent habit.

## Indication for a stoma in the case of colo-rectal anastomosis leak

Interestingly, regarding the decision to proceed with stoma, surgeons are actually split in two halves, with 217 (52.4%) relying of clinical symptoms, whereas the remaining ones considering AL size (percentage of anastomosis’ circumference). Interestingly, less experienced surgeons mostly decide based on leakage size compared to more experienced ones (*p* = 0.056). Probably, those latter ones’ experience, other than the recent introduction of various endoscopic techniques, tools and stents, make experienced colleagues more and more conservative even in the case of major leak, if patients are not severely symptomatic.

### Stoma technique in case of colo-rectal anastomotic leak

The vast majority (353/414; 85.3%) of surgeons prefer ileostomy as salvage procedure in the case of a colo-rectal AL. Interestingly enough, a laparoscopy-assisted stoma is an increasingly accepted approach among colo-rectal surgeons, as it is used by 83.4% (345/414) of colleagues, including ileal and colonic stomas.

### Indication for Hartmann procedure in the case of colo-rectal anastomosis leak

Interestingly, only 1.9% (8/414) of surgeon systematically opt for a Hartmann procedure in the case of colo-rectal anastomosis’ leak. On the contrary, almost 2/3 decides based on clinical symptoms (261/414; 63.1%), and 125/414 (30.2%) based on leak’s size. Similarly to what reported for the decision to perform a stoma, the severity of clinical symptoms lead the surgical management of colo-rectal anastomosis’ leak in most cases nowadays.

#### Indication for an endoscopic procedure in the case of colo-rectal anastomosis leak

Endoscopy has seemingly entered clinical practice, as less than 20% (80/414) of surgeons does not include endoscopy among colo-rectal anastomosis’ leak treatment options. “Sponge” (56.0%), clips (17.4%) and endorectal suturing (7.3%) are proposed as “endoscopic managements”, showing the first one as the preferred endoscopic approach by far. Even if no statistical analysis has been performed in this regard, interestingly, the placement of clips is seemingly considered more effective as non-operative management alone, as more than 70% (51/72; 70.8%) of patients undergoing endoscopic clipping do not undergo stoma, whereas sponge placement and suturing is proposed in association with ileostomy/colostomy in 55.2% (128/232) and 70% (21/30) of cases, respectively.

## Conclusions

The present survey represents an effort to define how colo-rectal leak prevention and management are evolving and how recently introduced techniques/tools and guidelines/recommendations have spread in surgeons’ practice.

Idropneumatic test and “donuts’” check are universally performed after left-sided procedures, whereas ICG test is rapidly spreading to check anastomotic stumps’ blood supply as it has already entered 2/3 of surgeons’ routine.

Drain placement is still largely adopted by most surgeons, with a decreasing rate from right (~ 50%) to left colectomy (~ 70%) to anterior resection (~ 90%), mostly ignoring ERAS recommendations.

A conservative management in clinically non-severe cases is spreading, as most surgeons’ management is led by leak’s symptoms and not size, both concerning the decision to proceed with protective right-sided stoma or Hartmann procedure, with surgeon’s experience playing a role in such an attitude.

Operative endoscopy, in particular endoscopic sponge placement, has definitely entered clinical practice as management option for over 80% of surgeons.

**Table 1:** Questions of the survey

General Information, personal experience and workplace workload in colorectal surgery	Q1	Gender
	Q2	Years of experience
	Q3	Workplace activity (colorectal resections per year)
	Q4	Setting of workplace
	Q5	Hospital with emergency (ER, emergency surgery) activities (Y/N)
	Q6	Hospital with intensive care unit (Y/N)
	Q7	Hospital with interventional radiology (Y/N)
	Q8	Workplace region in Italy
	Q9	Number of colectomies performed as operating surgeon
	Q10	Number of laparoscopic procedures performed as operating surgeon
Technique of low anastomosis	Q11	Ultra-low anastomosis (≤2 cm from the dentate line): which surgical technique?
Intraoperative “Tricks & Checks” for Leak Prevention	Q12	Prevention of colorectal fistula: which strategy?
	Q13	Prevention of colorectal fistula: which intraoperative leak test?
Indication and technique for stoma	Q14	Protective ileostomy/colostomy after ‘ideal’ mechanical anterior resection (Knight–Griffen technique)
	Q15	After anterior resection with mechanical colorectal anastomosis (Knight–Griffen), a ostomy is planned. Which technique?
	Q16	After anterior resection with mechanical colorectal anastomosis (Knight–Griffen), beyond the distance from the anal verge, which other factors influence the decision to perform a protective ileostomy/colostomy?
Drain management	Q17	Placement of drain(s) after right hemicolectomy
	Q18	Placement of drain(s) after left hemicolectomy
	Q19	Placement of drain(s) after anterior resection of the rectum
Leak Management (in the absence of stump ischemia, in an immunocompetent patient in good general condition)	Q20	Ileo-colic anastomosis with a paucisymptomatic clinically drained leak
	Q21	Reoperation for an ileo-colic anastomotic leak up to 2 cm with localized peritonitis
	Q22	Colorectal anastomosis with a paucisymptomatic clinically drainded leak
	Q23	Colorectal anastomosis with a drained colorectal leak: when should a diverting ileo-colostomy be considered?
	Q24	Leak on postoperative day 6 after laparoscopic anterior resection of the rectum with mechanical colorectal anastomosis (Knight-Griffen). A salvage ostomy is planned. Which technique should be used?
	Q25	Colorectal anastomosis with a drained leak: when should a Hartmann’s procedure be considered?
	Q26	Colorectal anastomosis with a drained leak involving up to one-third of the circumference: when should endoscopic treatment be considered?

Q=Question; Y/N = Yes/No

**Table 2:** Setting of the hospital

Question	Answer	No.	(%)
Type of hospital	Private hospital	2	0.5
	Semi-private hospital	20	4.8
	Public non-University hospital	194	46.9
	University hospital	198	47.8
No. of colectomies per year	0–50	275	66.4
	51–100	73	17.6
	> 100	66	16.0
Emergency activities	Yes	377	91.1
	No	37	8.9
Interventional radiology unit	Yes	321	77.5
	No	93	22.5
Intensive care unit	Yes	400	96.6
	No	14	3.4

No.=Number

**Table 3:** Working regions of respondents

Region	Survey participants	
	No.	%
Abruzzo	10	2.4
Basilicata	1	0.2
Calabria	4	1.0
Campania	29	7.0
Emilia-Romagna	66	15.9
Friuli-Venezia Giulia	6	1.5
Lazio	50	12.1
Liguria	17	4.1
Lombardia	74	17.9
Marche	4	1.0
Molise	8	1.9
Piemonte	30	7.2
Puglia	19	4.6
Sardegna	10	2.4
Sicilia	8	1.9
Toscana	45	10.9
Trentino-Alto Adige	3	0.7
Umbria	4	1.0
Valle d’Aosta	2	0.5
Veneto	24	5.8
Total	414	100

No.=Number

**Table 4:** Surgeon’s experience

Question	Answer	No.	%
Years of surgical experience	Resident	115	27.8
	< 5 y	126	30.4
	From 6 to 10 y	51	12.3
	> 10 y	122	29.5
Colectomies performed as operating surgeon (No.)	0–50	275	66.4
	51–200	73	17.6
	> 200	66	16.0
Laparoscopic procedures performed as operating surgeon (No.)	0–50	227	54.8
	51–200	89	21.5
	> 200	98	23.7

No.=Number; y=Years

**Table 5:** Technique of low Anastomosis

Question	Answer	Total (No. 414)		Surgeon’s no. of performed colectomies				p
		No.	%	0-50 (No. 275)		>50 (No. 139)		
				No.	%	No.	%	
Ultra-low anastomosis (≤2 cm from the dentate line): which surgical technique?	Manual colo-anal anastomosis	182	44.0	130	47.3	52	37.4	0.391
	Knight-Griffen (double-stapling)	155	37.4	99	36.0	56	40.3	
	Pull-through (various approaches)	36	8.7	21	7.6	15	10.8	
	TTSS	33	8.0	20	7.3	13	9.3	
	Other	8	1.9	5	1.8	3	2.2	

**Table 6:** Intraoperative “Tricks & Checks” in leak prevention

Question	Answer	Total (No. 414)		Surgeon’s no. of performed colectomies				p
		No.	%	0-50 (No. 275)		>50 (No. 139)		
				No.	%	No.	%	
Prevention of colorectal fistula: which strategy?	Nothing	161	38.9	98	35.6	63	45.3	0.098*
	Anastomosis’ reinforcement suture	123	29.7	88	32	35	25.2	
	Any technique to prevent “dog-ears”	90	21.7	65	23.6	25	18.0	
	Glue	23	5.6	18	6.6	5	3.6	
	Other	17	4.1	6	2.2	11	7.9	
Prevention of colorectal fistula: which intraoperative leak test?^#^	Hydropneumaticleaktest	393	94.9	261	94.9	132	95.0	0.866*
	Rings’ check	364	87.9	240	87.3	124	89.2	
	ICG	256	61.8	162	58.9	94	67.6	
	Other	18	4.3	7	2.5	11	7.9	
	Nothing	3	0.7	2	0.7	1	0.7	

**Table 7:** Indication and technique for stoma

Question	Answer	Total (No. 414)		Surgeon’s no. of performed colectomies				p
		No.	%	0-50 (No. 275)		>50 (No. 139)		
				No.	%	No.	%	
Protective ileo/colostomy after ‘ideal’ mechanical anterior resection (Knight–Griffen technique)	Never	2	0.5	0	0.0	2	1.4	0.023
	Non-systematic (depending on local/patient conditions)	217	52.4	157	57.1	60	43.2	
	Always (systematic <12 cm)	25	6.0	15	5.5	10	7.2	
	Systematic <4 cm	107	25.9	70	25.4	37	26.6	
	Systematic <8 cm	63	15.2	33	12.0	30	21.6	
After anterior resection with mechanical colorectal anastomosis (Knight–Griffen), I decide to perform a protective stoma. Which technique?	Left colostomy	11	2.7	7	2.5	4	2.9	0.212
	Ileostomy 15 cm from ICV	220	53.1	147	53.5	73	52.5	
	Ileostomy 30 cm from ICV	169	40.8	116	42.2	53	38.1	
	Right transversostomy	9	2.2	3	1.1	6	4.3	
	Other	5	1.2	2	0.7	3	2.2	
After anterior resection with mechanical colorectal anastomosis (Knight–Griffen), beyond the distance from the anal verge, which other factors influence the decision to perform a protective ileo/colostomy?^**#**^	Other relevant comorbidities (cardiac, respiratory, etc.)	256	61.8	161	58.5	95	68.3	0.617*
	BMI	204	49.3	136	49.5	68	48.9	
	Local conditions (tissue inflammation, etc.)	350	84.5	238	86.5	112	80.6	
	Preoperative RT	348	84.1	229	83.3	119	85.6	
	Quality of anastomosis (number of firings with linear stapler, etc.)	291	70.3	194	70.5	97	69.8	
	Type II Diabetes Mellitus	168	40.6	158	57.5	10	7.2	
	Smoking	126	30.4	76	27.6	50	36.0	

**Table 8:** Drain management

Question	Answer	Total (No. 414)		Surgeon’s no. of performed colectomies				p
		No.	%	0-50 (No. 275)		>50 (No. 139)		
				No.	%	No.	%	
Placement of drain(s) after right hemicolectomy	No drain	152	36.7	95	34.5	57	41.0	0.401*
	One, retroanastomotic	76	18.4	57	20.7	19	13.7	
	One, in the right parietocolic gutter	72	17.4	45	16.4	27	19.4	
	No drain if the patient follows the ERAS protocol	67	16.2	49	17.8	18	13.0	
	One, in the pelvic cavity	29	7.0	17	6.2	12	8,6	
	Two	12	2.9	8	2.9	4	2.9	
	Other	6	1.4	4	1.5	2	1.4	
Placement of drain(s) after left hemicolectomy	One, in the pelvic cavity	203	49.0	142	51.6	61	43.9	0.044*
	No drain	74	17.9	37	13.5	37	26.6	
	One, in the left parietocolic gutter	53	12.8	38	13.8	15	10.8	
	No drain if the patient follows the ERAS protocol	37	8.9	24	8.7	13	9.4	
	One, if the patient has particular comorbidities (diabetes, obesity, etc.)	22	5.3	16	5.8	6	4.3	
	Two	21	5.1	15	5.5	6	4.3	
	Other	4	1.0	3	1.1	1	0.7	
Placement of drain(s) after anterior resection of the rectum	One, in the pelvic cavity	305	73.7	203	73.8	102	73.4	0.714*
	Two	49	11.8	33	12.0	16	11.5	
	No drain	24	5.8	14	5.1	10	7.2	
	No drain if the patient follows the ERAS protocol	14	3.4	10	3.6	4	2.9	
	One, if the patient has particular comorbidities (diabetes, obesity, etc.)	13	3.1	11	4.0	2	1.4	
	Other	5	1.2	1	0.4	4	2.9	
	One, in the left parietocolic gutter	4	1.0	3	1.1	1	0.7	

**Table 9:** Leak management

Question	Answer	Total (No. 414)		Surgeon’s no. of performed colectomies				p
		No.	%	0-50 (No. 275)		>50 (No. 139)		
				No.	%	No.	%	
Ileo-colic anastomosis with a clinically paucisymptomatic, drained leak	Conservative approach (observation and CT)	304	73.4	211	76.7	93	66.9	0.023*
	Laparoscopy	66	15.9	38	13.8	28	20.1	
	Colonoscopy (e.g. endoscopic procedures)	26	6.3	18	6.6	8	5.8	
	Laparotomy	14	3.4	5	1.8	9	6.5	
	Other	4	1.0	3	1.1	1	0.7	
Reoperation for ileo-colic anastomotic leak up to 2 cm with localized peritonitis	Ileocolic reanastomosis without ileostomy	149	36.0	87	31.6	62	44.6	0.104*
	Ileocolic reanastomosis with ileostomy or ileo/colostomy	122	29.5	89	32.4	33	23.7	
	Raphia with ileostomy	98	23.7	69	25.1	29	20.8	
	Terminal ileostomy or ileo/colostomy	30	7.2	21	7.6	9	6.5	
	Raphia alone (without stoma)	10	2.4	7	2.6	3	2.2	
	Other	5	1.2	2	0.7	3	2.2	
Colorectal anastomosis with a clinically paucisymptomatic, drained leak	Conservative approach (observation and CT)	273	65.9	183	66.6	90	64.7	0.894*
	Colonoscopy (e.g. endoscopic procedures)	63	15.2	43	15.6	20	14.4	
	Laparoscopy ± Ileo/colostomy	55	13.3	36	13.1	19	13.7	
	Ileo/colostomy with open access	11	2.7	7	2.5	4	2.9	
	Other	10	2.4	5	1.8	5	3.6	
	Laparotomy	2	0.5	1	0.4	1	0.7	
Colorectal anastomosis with a drained leak: when should a diverting ileo-colostomy be considered?	Depends on the clinic	217	52.4	132	48.0	85	61.1	0.056*
	Leak size > 1⁄3 of the circumference	63	15.2	49	17.8	14	10.1	
	Systematic	56	13.5	40	14.5	16	11.5	
	Leak size > 1/4 of the circumference	38	9.2	27	9.8	11	7.9	
	Leak size > 1/2 of the circumference	34	8.2	26	9.5	8	5.8	
	Other	6	1.5	1	0.4	5	3.6	
Salvage diverting stoma for POD 6 leak after LAR with mechanical colorectal anastomosis (Knight-Griffen): Which technique should be used?	Ileostomy 15 cm from ICV, with LA	171	41.3	115	41.8	56	40.3	0.133*
	Ileostomy 30 cm from ICV, with LA	134	32.4	91	33.1	43	30.9	
	Right transversostomy, with LA	40	9.7	19	6.9	21	15.1	
	Ileostomy 15 cm from ICV, with OA	26	6.3	19	6.9	7	5.0	
	Ileostomy 30 cm from ICV, with OA	22	5.3	18	6.5	4	2.9	
	Other	12	2.9	7	2.5	5	3.6	
	Right transversostomy, with OA	9	2.2	6	2.3	3	2.2	
Colorectal anastomosis’ drained leak: when should Hartmann’s procedure be considered?	Depending on the clinical symptoms	261	63.1	178	64.7	83	59.7	0.365*^#^
	Leak size > 1/2 of the circumference	81	19.6	58	21.1	23	16.6	
	Leak size > 1/3 of the circumference	44	10.6	25	9.1	19	13.7	
	Other	20	4.8	8	2.9	12	8.6	
	Systematically	8	1.9	6	2.2	2	1.4	
Colorectal anastomosis’ drained leak involving up to one-third of the circumference: when should an endoscopic treatment be considered?	Intracavitary sponge (e.g. endosponge) with stoma	128	30.9	85	30.9	43	30.9	0.820*
	Intracavitary sponge (e.g endosponge) without stoma	104	25.1	72	26.1	32	23.0	
	Endoscopic approach is not considered	80	19.3	48	17.5	32	23.0	
	Clips (e.g. Ovesco) without stoma	51	12.3	33	12.0	18	12.9	
	Clips (e.g. Ovesco) with stoma	21	5.1	15	5.5	6	4.4	
	Endoscopic suture with stoma	21	5.1	15	5.5	6	4.4	
	Endoscopic suture without stoma	9	2.2	7	2.5	2	1.4	

## Supplementary Information

Below is the link to the electronic supplementary material.

Supplementary Material 1

## Data Availability

Data archiving is not mandated but data will be made available on reasonable request.

## References

[1] Rennie O, Sharma M, Helwa N (2024) Colorectal anastomotic leakage: a narrative review of definitions, grading systems, and consequences of leaks. Front Surg 11:1371567. 10.3389/fsurg.2024.1371567. (in eng) 38756356 10.3389/fsurg.2024.1371567 PMC11097957

[2] Telem DA, Chin EH, Nguyen SQ, Divino CM (2010) Risk factors for anastomotic leak following colorectal surgery: a case-control study, (in eng). Arch Surg 145(4):371–376 discussion 376, Apr. 10.1001/archsurg.2010.40 20404288 10.1001/archsurg.2010.40

[3] Zarnescu EC, Zarnescu NO, Costea R (2021) Updates of risk factors for anastomotic leakage after colorectal surgery, (in eng), *Diagnostics (Basel)*, vol. 11, no. 12, Dec 17 10.3390/diagnostics11122382 10.3390/diagnostics11122382 PMC8700187 34943616

[4] Tonini V, Zanni M Impact of anastomotic leakage on long-term prognosis after colorectal cancer surgery, (in eng). World J Gastrointest Surg, 15, 5, pp. 745–756, May 27 2023, 10.4240/wjgs.v15.i5.745 10.4240/wjgs.v15.i5.745 PMC10277951 37342854

[5] Koedam TWA et al (2022) Oncological outcomes after anastomotic leakage after surgery for colon or rectal cancer: increased risk of local recurrence, (in eng). Ann Surg 275(2):e420–e. 10.1097/sla.0000000000003889 32224742 10.1097/SLA.0000000000003889

[6] Frasson M et al (2015) Risk factors for anastomotic leak after colon resection for cancer: multivariate analysis and nomogram from a multicentric, prospective, national study with 3193 patients, (in eng), *Ann Surg*, vol. 262, no. 2, pp. 321 – 30, Aug 10.1097/sla.0000000000000973 10.1097/SLA.0000000000000973 25361221

[7] He J, He M, Tang JH, Wang XH (2023) Anastomotic leak risk factors following colon cancer resection: a systematic review and meta-analysis, (in eng), *Langenbecks Arch Surg*, vol. 408, no. 1, p. 252, Jun 29 10.1007/s00423-023-02989-z 10.1007/s00423-023-02989-z 37386211

[8] Park JS et al (2013) Multicenter analysis of risk factors for anastomotic leakage after laparoscopic rectal cancer excision: the Korean laparoscopic colorectal surgery study group, (in eng), *Ann Surg*, vol. 257, no. 4, pp. 665 – 71, Apr 10.1097/SLA.0b013e31827b8ed9 10.1097/SLA.0b013e31827b8ed9 23333881

[9] Arezzo A et al (Jul 2019) The REAL (REctal Anastomotic Leak) score for prediction of anastomotic leak after rectal cancer surgery, (in eng). Tech Coloproctol 23:649–663. 10.1007/s10151-019-02028-4 10.1007/s10151-019-02028-4 31240416

[10] Venn ML, Hooper RL, Pampiglione T, Morton DG, Nepogodiev D, Knowles CH (Jul 18 2023) Systematic review of preoperative and intraoperative colorectal Anastomotic Leak Prediction Scores (ALPS), (in eng). BMJ Open 13(7):e073085. 10.1136/bmjopen-2023-073085 10.1136/bmjopen-2023-073085 PMC10357690 37463818

[11] Favuzza J (2021) Risk factors for anastomotic leak, consideration for proximal diversion, and appropriate use of drains, (in eng), *Clin Colon Rectal Surg*, vol. 34, no. 6, pp. 366–370, Nov 10.1055/s-0041-1735266 10.1055/s-0041-1735266 PMC8610643 34853556

[12] Tsalikidis C et al (2023) Predictive Factors for Anastomotic Leakage following colorectal cancer surgery: where are we and where are we going? (in eng), *Curr Oncol*, vol. 30, no. 3, pp. 3111–3137, Mar 7 10.3390/curroncol30030236 10.3390/curroncol30030236 PMC10047700 36975449

[13] De Nardi P et al (2020) Intraoperative angiography with indocyanine green to assess anastomosis perfusion in patients undergoing laparoscopic colorectal resection: results of a multicenter randomized controlled trial, (in eng), *Surg Endosc*, vol. 34, no. 1, pp. 53–60, Jan 10.1007/s00464-019-06730-0 10.1007/s00464-019-06730-0 30903276

[14] Jayne D et al (2025) Sep., Intraoperative fluorescence angiography with indocyanine green to prevent anastomotic leak in rectal cancer surgery (IntAct): an unblinded randomised controlled trial, (in eng), *Lancet Gastroenterol Hepatol*, vol. 10, no. 9, pp. 806–817. 10.1016/s2468-1253(25)00101-3 10.1016/S2468-1253(25)00101-3 40690925

[15] Ho SYA, Muthiah VK, Tay KV (2024) Comparing surgical outcomes of powered versus manual surgical staplers: a systematic review and meta-analysis, (in eng), *Langenbecks Arch Surg*, vol. 409, no. 1, p. 331, Oct 31 10.1007/s00423-024-03490-x 10.1007/s00423-024-03490-x 39480563

[16] Scardino A et al (Sep 27 2024) Effect of powered circular stapler in colorectal anastomosis after left-sided colic resection: systematic review and meta-analysis, (in eng). Int J Colorectal Dis 39(1):152. 10.1007/s00384-024-04729-1 10.1007/s00384-024-04729-1 PMC11436432 39331160

[17] Fiorillo C et al (Mar 4 2025) Circular staplers and anastomotic leakage in colorectal surgery: meta-analysis. BJS Open 9(2) (in eng). 10.1093/bjsopen/zrae170 10.1093/bjsopen/zrae170 PMC11979101 40200762

[18] Griffen FD, Knight CD, Sr. JM, Whitaker, Knight CD Jr. (1990) The double stapling technique for low anterior resection. Results, modifications, and observations, (in eng), *Ann Surg*, vol. 211, no. 6, pp. 745 – 51; discussion 751-2, Jun 10.1097/00000658-199006000-00014 10.1097/00000658-199006000-00014 PMC1358129 2357137

[19] Yue Y et al (2024) Effectiveness of anastomotic reinforcement sutures in reducing anastomotic leakage risk after laparoscopic rectal cancer surgery: a pooled and integration analysis. Front Oncol 14:1337870. 10.3389/fonc.2024.1337870. (in eng) 38894871 10.3389/fonc.2024.1337870 PMC11183793

[20] Zhang T et al (2023) Clinical efficacy of anastomotic reinforcement suture in preventing anastomotic leakage after rectal cancer surgery: a systematic review and meta-analysis, (in eng), *Langenbecks Arch Surg*, vol. 408, no. 1, p. 322, Aug 18., 10.1007/s00423-023-03058-1 10.1007/s00423-023-03058-1 37594605

[21] Spinelli A et al (Dec 2021) Transanal Transection and Single-Stapled Anastomosis (TTSS): a comparison of anastomotic leak rates with the double-stapled technique and with transanal total mesorectal excision (TaTME) for rectal cancer, (in eng). Eur J Surg Oncol 47(12):3123–3129. 10.1016/j.ejso.2021.08.002 10.1016/j.ejso.2021.08.002 34384655

[22] Picciariello A, Gravante G, Annicchiarico A, Melcarne R, Vincenti L (2025) Comprehensive evaluation of reinforcement strategies for anastomotic leak prevention in rectal cancer surgery: an umbrella review of meta-analyses, (in eng). Updates Surg Aug 16. 10.1007/s13304-025-02366-z 10.1007/s13304-025-02366-z 40819153

[23] Gustafsson UO et al (Mar 2019) Guidelines for perioperative care in elective colorectal surgery: enhanced recovery after surgery (ERAS(^®^)) Society Recommendations: 2018, (in eng). World J Surg 43(3):659–695. 10.1007/s00268-018-4844-y 10.1007/s00268-018-4844-y 30426190

[24] Phitayakorn R et al (Jun 2008) Standardized algorithms for management of anastomotic leaks and related abdominal and pelvic abscesses after colorectal surgery, (in eng). World J Surg 32(6):1147–1156. 10.1007/s00268-008-9468-1 10.1007/s00268-008-9468-1 18283511

[25] Cuccurullo D et al (2015) Relaparoscopy for management of postoperative complications following colorectal surgery: ten years experience in a single center, (in eng), *Surg Endosc*, vol. 29, no. 7, pp. 1795 – 803, Jul 10.1007/s00464-014-3862-6 10.1007/s00464-014-3862-6 25294542

[26] Lee CM et al (Apr 2015) Laparoscopic versus open reintervention for anastomotic leakage following minimally invasive colorectal surgery, (in eng). Surg Endosc 29(4):931–936. 10.1007/s00464-014-3755-8 10.1007/s00464-014-3755-8 25060688

[27] Sevim Y, Celik SU, Yavarifar H, Akyol C Minimally invasive management of anastomotic leaks in colorectal surgery, (in eng). World J Gastrointest Surg, 8, 9, pp. 621–626, Sep 27 2016, 10.4240/wjgs.v8.i9.621 10.4240/wjgs.v8.i9.621 PMC5037335 27721925

[28] Thomas MS, Margolin DA (2016) Management of colorectal anastomotic leak, (in eng), *Clin Colon Rectal Surg*, vol. 29, no. 2, pp. 138 – 44, Jun 10.1055/s-0036-1580630 10.1055/s-0036-1580630 PMC4882170 27247539

[29] Şandra-Petrescu F, Tzatzarakis E, Kähler G, Reissfelder C, Herrle F (Oct 2021) Management of colorectal anastomotic leakage using endoscopic negative pressure therapy with or without protective ostomy: a retrospective study, (in eng). Int J Colorectal Dis 36(10):2261–2269. 10.1007/s00384-021-04011-8 10.1007/s00384-021-04011-8 PMC8426235 34455472

[30] DiMaio CJ et al (2012) Covered esophageal self-expandable metal stents in the nonoperative management of postoperative colorectal anastomotic leaks, (in eng), *Gastrointest Endosc*, vol. 76, no. 2, pp. 431-5, Aug 10.1016/j.gie.2012.03.1393 10.1016/j.gie.2012.03.1393 22817797

[31] Shalaby M, Emile S, Elfeki H, Sakr A, Wexner SD, Sileri P (2019) Systematic review of endoluminal vacuum-assisted therapy as salvage treatment for rectal anastomotic leakage, (in eng), *BJS Open*, vol. 3, no. 2, pp. 153–160, Apr 10.1002/bjs5.50124 10.1002/bjs5.50124 PMC6433422 30957061

[32] Heiss MM et al (2024) Treatment of anastomotic leak in colorectal surgery by endoluminal vacuum therapy with the VACStent avoiding a stoma - a pilot study, (in eng), *Langenbecks Arch Surg*, vol. 409, no. 1, p. 234, Jul 31 10.1007/s00423-024-03426-5 10.1007/s00423-024-03426-5 PMC11291571 39083099

[33] Matthiessen P, Hallböök O, Rutegård J, Simert G, Sjödahl R (2007) Defunctioning stoma reduces symptomatic anastomotic leakage after low anterior resection of the rectum for cancer: a randomized multicenter trial, (in eng), *Ann Surg*, vol. 246, no. 2, pp. 207 – 14, Aug 10.1097/SLA.0b013e3180603024 10.1097/SLA.0b013e3180603024 PMC1933561 17667498

[34] Gu WL, Wu SW (Jan 24 2015) Meta-analysis of defunctioning stoma in low anterior resection with total mesorectal excision for rectal cancer: evidence based on thirteen studies, (in eng). World J Surg Oncol 13:9. 10.1186/s12957-014-0417-1 10.1186/s12957-014-0417-1 PMC4311499 25617234

[35] Goligher JC, Graham NG, De Dombal FT (1970) Anastomotic dehiscence after anterior resection of rectum and sigmoid, (in eng), *Br J Surg*, vol. 57, no. 2, pp. 109 – 18, Feb 10.1002/bjs.1800570208 10.1002/bjs.1800570208 5467147

[36] Morgenstern L, Yamakawa T, Ben-Shoshan M, Lippman H (Jan 1972) Anastomotic leakage after low colonic anastomosis. Clinical and experimental aspects, (in eng). Am J Surg 123(1):104–109. 10.1016/0002-9610(72)90317-0 10.1016/0002-9610(72)90317-0 5058865

[37] Litchinko A et al (2024) Score prediction of anastomotic leak in colorectal surgery: a systematic review, (in eng), *Surg Endosc*, vol. 38, no. 4, pp. 1723–1730, Apr 10.1007/s00464-024-10705-1 10.1007/s00464-024-10705-1 PMC10978556 38418633

[38] Saur NM, Paulson EC (May 2019) Operative management of Anastomotic leaks after colorectal surgery, (in eng). Clin Colon Rectal Surg 32(3):190–195. 10.1055/s-0038-1677025 10.1055/s-0038-1677025 PMC6494610 31061649

[39] Tsai YY, Chen WT (Dec 2019) Management of anastomotic leakage after rectal surgery: a review article, (in eng). J Gastrointest Oncol 10:1229–1237. 10.21037/jgo.2019.07.07 10.21037/jgo.2019.07.07 PMC6955017 31949944

[40] Vogel JD et al (Feb 1 2022) The american society of colon and rectal surgeons clinical practice guidelines for the management of colon cancer, (in eng). Dis Colon Rectum 65(2):148–177. 10.1097/dcr.0000000000002323 10.1097/DCR.0000000000002323 34775402

[41] Tzanis AA et al (2025) A systematic review, meta-analysis and GRADE assessment of the evidence on complete mesocolic excision for right-sided colon cancer with SAGES and ESCP participation, (in eng), *Surg Endosc*, vol. 39, no. 6, pp. 3466–3473, Jun 10.1007/s00464-025-11749-7 10.1007/s00464-025-11749-7 40325243

[42] Huo B et al (Feb 2025) Surgical management of complicated diverticulitis: systematic review and individual patient data network meta-analysis: An EAES/ESCP collaborative project, (in eng). Surg Endosc 39(2):699–715. 10.1007/s00464-024-11457-8 10.1007/s00464-024-11457-8 39733170

[43] Sammour T et al (2017) Validation of an online risk calculator for the prediction of anastomotic leak after colon cancer surgery and preliminary exploration of artificial intelligence-based analytics, (in eng), *Tech Coloproctol*, vol. 21, no. 11, pp. 869–877, Nov 10.1007/s10151-017-1701-1 10.1007/s10151-017-1701-1 29080956

[44] Yang SU, Park EJ, Baik SH, Lee KY, Kang J (2019) Modified colon leakage score to predict Anastomotic leakage in patients who underwent left-sided colorectal surgery, (in eng), *J Clin Med*, vol. 8, no. 9, Sep 12 10.3390/jcm8091450 10.3390/jcm8091450 PMC6780090 31547283

[45] Zhang Z et al (2022) A nomogram to predict the risk of colorectal anastomotic leakage combining inflammatory-nutritional and abdominal aorta calcium index. Front Surg 9:p1008448. 10.3389/fsurg.2022.1008448. (in eng) 10.3389/fsurg.2022.1008448 PMC9852538 36684195

[46] Asteria CR et al (2008) Anastomotic leaks after anterior resection for mid and low rectal cancer: survey of the Italian Society of Colorectal Surgery, (in eng), *Tech Coloproctol*, vol. 12, no. 2, pp. 103 – 10, Jun 10.1007/s10151-008-0407-9 10.1007/s10151-008-0407-9 18545882

[47] Spinelli A et al (2020) Italian multi-society modified Delphi consensus on the definition and management of anastomotic leakage in colorectal surgery, (in eng), *Updates Surg*, vol. 72, no. 3, pp. 781–792, Sep 10.1007/s13304-020-00837-z 10.1007/s13304-020-00837-z 32613380

[48] Șandra-Petrescu F et al (Jul 27 2023) Management of Anastomotic leakage after colorectal resection: survey among the German CHIR-Net centers, (in eng). J Clin Med 12(15). 10.3390/jcm12154933 10.3390/jcm12154933 PMC10419945 37568336

[49] Rajabaleyan P et al (Oct 2024) Early warning model to detect anastomotic leakage following colon surgery: a clinical observational study, (in eng). Ann Coloproctol 40(5):431–439. 10.3393/ac.2023.00745.0106 10.3393/ac.2023.00745.0106 PMC11532379 39376121

[50] Eysenbach G (2004) Improving the quality of Web surveys: the checklist for reporting results of internet E-Surveys (CHERRIES). J Med Internet Res 6(3):e34 15471760 10.2196/jmir.6.3.e34 PMC1550605

[51] Bianco F, Novi A, Incollingo P, Gallo G, Grassia S (Jun 2022) The short stump and high anastomosis pull-through procedure for delayed coloanal anastomosis with no protective stoma for low rectal cancer - a video vignette, (in eng). Colorectal Dis 24(6):801–802. 10.1111/codi.16087 10.1111/codi.16087 35133705

[52] Biondo S et al (2024) Long-Term Results of 2-Stage Turnbull-Cutait Pull-Through Coloanal Anastomosis for Low Rectal Cancer: a randomized clinical trial, (in eng), *JAMA Surg*, vol. 159, no. 9, pp. 990–996, Sep 1., 10.1001/jamasurg.2024.2262 10.1001/jamasurg.2024.2262 PMC11238068 38985480

[53] Cutait DE, Figliolini FJ (Sep-Oct 1961) A new method of colorectal anastomosis in abdominoperineal resection, (in eng). Dis Colon Rectum 4:335–342. 10.1007/bf02627230 10.1007/BF02627230 13882795

[54] Kirwan WO, Turnbull RB Jr., Fazio VW, Weakley FL (Oct 1978) Pullthrough operation with delayed anastomosis for rectal cancer, (in eng). Br J Surg 65(10):695–698. 10.1002/bjs.1800651008 10.1002/bjs.1800651008 709078

[55] Rutegård M et al (Feb 2025) SELective defunctioning Stoma Approach in low anterior resection for rectal cancer (SELSA): protocol for a prospective study with a nested randomized clinical trial investigating stoma-free survival without major LARS following total mesorectal excision, (in eng). Colorectal Dis 27(2):e70009. 10.1111/codi.70009 10.1111/codi.70009 PMC11780343 39887540

[56] Davis BR, Valente MA, Goldberg JE, Lightner AL, Feingold DL, Paquette IM (Oct 1 2022) The American Society of Colon and Rectal Surgeons Clinical Practice Guidelines for Ostomy Surgery, (in eng). Dis Colon Rectum 65(10):1173–1190. 10.1097/dcr.0000000000002498 10.1097/DCR.0000000000002498 35616386

[57] Yang S, Tang G, Zhang Y, Wei Z, Du D (May 8 2024) Meta-analysis: loop ileostomy versus colostomy to prevent complications of anterior resection for rectal cancer, (in eng). Int J Colorectal Dis 39(1):68. 10.1007/s00384-024-04639-2 10.1007/s00384-024-04639-2 PMC11076370 38714581

[58] Yang YW et al (2024) Protective loop ileostomy or colostomy? A risk evaluation of all common complications, (in eng), *Ann Coloproctol*, vol. 40, no. 6, pp. 580–587, Dec 10.3393/ac.2022.00710.0101 10.3393/ac.2022.00710.0101 PMC11701458 36702474

[59] Sica GS et al (Jul 6 2024) Gastrointestinal functions after laparoscopic right colectomy with intracorporeal anastomosis: a pilot randomized clinical trial on effects of abdominal drain, prolonged antibiotic prophylaxis, and D3 lymphadenectomy with complete mesocolic excision, (in eng). Int J Colorectal Dis 39(1):102. 10.1007/s00384-024-04657-0 10.1007/s00384-024-04657-0 PMC11227461 38970713

[60] Brunner M, Bondartschuk K, Denz A, Weber GF, Grützmann R, Krautz C (Jul 12 2025) The role of intraabdominal drain placement in minimal-invasive right hemicolectomy with complete mesocolic excision - a propensity score matched single center analysis, (in eng). Int J Colorectal Dis 40(1):156. 10.1007/s00384-025-04948-0 10.1007/s00384-025-04948-0 PMC12254058 40646393

[61] Vignali A, Elmore U, Aleotti F, Roberto D, Parise P, Rosati R (2021) Re-laparoscopy in the treatment of anastomotic leak following laparoscopic right colectomy with intracorporeal anastomosis, (in eng), *Surg Endosc*, vol. 35, no. 11, pp. 6173–6178, Nov 10.1007/s00464-020-08113-2 10.1007/s00464-020-08113-2 33104916

